# Lectures on the Diagnosis and Treatment of Diseases of the Lungs

**Published:** 1841-03-27

**Authors:** W. W. Gerhard


### MEDICAL EXAMINER.

DEVOTED TO MEDICINE, SURGERY, AND THE COLLATERAL SCIENCES.

No. 13.] PHILADELPHIA, SATURDAY, MARCH 27, 1841. [Vol. IV.

### LECTURESON THE DIAGNOSIS AND TREATMENT OF DISEASES OF THE LUNGS.

BY W. W. GERHARD, M. D.

### LECTURE XIII.—(*Continued.*)

#### PHTHISIS—PROGNOSIS.

The prognosis of phthisis is unfortunately quite clear in the large majority of cases: and when the disease is established, it is regarded as almost necessarily fatal. This prognosis must, however, be taken with some reservation, as the disease is in its nature essentially different in different stages, and cannot be said to be unavoidably fatal except when the disorganization of the lungs is much advanced, and the tuberculous degeneration of the whole economy is carried to a very high degree. In the earlier stages the disorder may terminate in recovery; and there is no doubt that it not unfrequently gets well, even when the local sign of the disorder, the deposit of tubercles themselves, is actually formed. But these are not the most frequent cases; for before any actual deposit of tubercle can take place, a very extensive alteration of the whole fluids has in all probability occurred, and the deterioration will be found to have reached that point which renders recovery rare, if not impracticable.

Although in many cases of phthisis the possibility of recovery is now generally admitted, this result is by no means probable, except when a number of favourable circumstances concur; for, as the causes of phthisis are for the most part very slow, but at the same time very powerful in their action, the disease cannot in many instances be materially influenced by remedies. It is therefore unfortunately true, that even when we foresee that the disease is approaching, or distinguish the first steps of the tuberculous formation, it cannot always be arrested,—but there are other cases in which the result is happily much more favourable. In order to distinguish these cases, we must bear in mind the circumstances already mentioned as complicating the progress of phthisis, or influencing its development. Of these the most important are a strong predisposition to phthisis, whether hereditary or acquired, and an exposure to circumstances known to favour the development of the disease. Individuals who present this constitutional tendency are those who offer the well-known signs of the scrofulous constitution known by the peculiar colour of the skin, and have especially the very dark or the light rosy complexion; when the disease is hereditary, the dark complexion is perhaps more frequent than the light, and the skin generally a dusky, earthy tint, or a dirty aspect, which is often almost peculiar to this disease. It is not always the case that those persons are thin and feeble,—some of them are stout and muscular, but feebleness of body increases the tendency to the tuberculous development, and we may make the same remark with still greater force of the fat, pale, tallow-like complexion of some individuals, especially women, who possess an hereditary tendency to phthisis; the latter class of patients generally offer an enlarged, fatty state of the liver, and the prognosis in this case becomes very unfavourable.

If exposure to the causes favouring tuberculous development cannot be prevented, the influence of them must be obvious enough, and will greatly increase the probability of an unfavourable issue. In all cases, therefore, in which any direct evidence of tuberculous deposit is conjoined with the hereditary tendency, or strongly disposing causes, the disease is most intractable; this accounts in a great measure for the more frequently fatal termination of phthisis in the crowded and impure wards of an hospital. If there be no local signs whatever, but merely that constitutional deterioration which, unless arrested, is sure to end in phthisis, our prognosis is different; these indeed are scarcely to be considered as true cases of consumption; they are so only in embryo, and may be often arrested by change of residence or other means.

The mode of development, and the early symptoms of phthisis, have also a considerable influence upon its termination. Some cases are unfavourable from the beginning, not merely from the strongly marked general symptoms, but because the local symptoms are known by experience to coincide with intractable forms of the disorder. The signs which may be regarded as impressing a favourable character upon the disease, are hæmoptysis, if occurring from time to time after exertion, and very moderate, and local inflammation of the lungs before tuberculous matter is deposited in any large quantity. Hæmoptysis, if extremely abundant, is not favourable, unless the patient should get perfectly well without irritative fever or further symptoms of tubercles, it then seems to relieve the lungs, and the disease is in general milder, and not unfrequently abates at an early period. But there is another form of hæmoptysis which often occurs long before the disease seems to be concentrated upon the lungs, and is perhaps rather referrible to a peculiar condition of the whole capillary system, than to any local mischief, this renders the prognosis much less favourable; it is the spitting of blood, which often continues for years, a mouthful or two at a time after coughing or very slight efforts, and is hardly noticed by the patient. It is most frequent in young women, and in those who offer a strong constitutional tendency to phthisis.

The local inflammation of the serous membranes, or of the serous tissue of the lungs, is a favourable sign, because the action which gives rise to the disease is here a positive, tangible one; and if we succeed in changing it, or modifying its progress before tubercles are formed, the disorder may be arrested much more easily than in the more constitutional cases. If the tuberculous matter be actually formed, but limited to small portions of the lung, the prognosis may still remain favourable to a certain extent,—that is, the disease will be slow, and in a few cases will terminate happily, notwithstanding a cavity is formed.

The least favourable local signs are those observed when the disease begins in a slow, insidious manner, by the trachea or larynx, which does not always call attention to the lungs, and the tuberculous degeneration proceeds in an unsuspected form. Not that chronic laryngitis is of itself necessarily fatal, but it certainly promotes the formation of tubercles; and when this point is once reached, the disease generally assumes a severe and unmanageable character. In these cases, too, the tubercles are often scattered widely through the lungs, and of course are of more mischief than if they are limited to a small space at the summit.

The prognosis of phthisis must be taken in a more extended sense than that of its ultimate termination: we have to decide in many cases whether the disease is to terminate speedily or slowly in death or recovery, this investigation leads us to the study of the varieties of phthisis in relation to its character.

*Duration of Phthisis.*—Although consumption of the lungs is, in the large majority of cases, a chronic disease, it is from time to time met with in an acute form; that is, it may prove fatal in a period of less than three months. This depends upon the rapid formation of gray granulations, or tuberculous infiltration in a large portion of the lungs. The disease is then attended with much fever, and the general tuberculous signs, as already mentioned, are extremely developed. In many instances, death does not take place so much from the pulmonary disorder, as from the coincident inflammations, or tuberculous deposition in other organs, especially in the serous membranes of the brain. But phthisis may become acute, when it begins in the ordinary chronic form, and the change is then rendered apparent by the rapid increase and severe character of the fever and sweats. Hence, although we know that the usual course of ordinary phthisis is slow, it is always possible that the type may change, and the termination may be hurried much more rapidly than usual.

Our prognosis in acute cases is directly dependent upon the diagnosis; for if we once recognise the disease as of the acute form, we can confidently state that its course will be probably a short and a fatal one. The duration of the ordinary variety of phthisis has been estimated by Dr. Louis to be about eighteen months; this is, perhaps, sufficiently near the truth,—but a large proportion of cases in hospital practice terminate in less than that period; in private practice the course of the disease is delayed so much by treatment, that the average duration of all cases, except the acute, is probably two years. The duration of consumption is greatly influenced by age: the disease is often acute in the young—rarely so in those more advanced in life; the female sex has a similar influence with childhood, so that the most frequent cases of acute phthisis are to be met with in young girls, a little after the age of puberty.

